# Supplementary material for: Enhancing the Antioxidant, Antibacterial, and Wound Healing Effects of Melaleuca alternifolia Oil by Microencapsulating It in Chitosan-Sodium Alginate Microspheres
Source: Nutrients. 2023 Mar 7;15(6):1319. doi: 10.3390/nu15061319 (PMC10051692; doi:10.3390/nu15061319)
Supplement: Supplementary file 1 [file nutrients-15-01319-s001.zip › nutrients-2251167-supplementary.pdf]

(a)

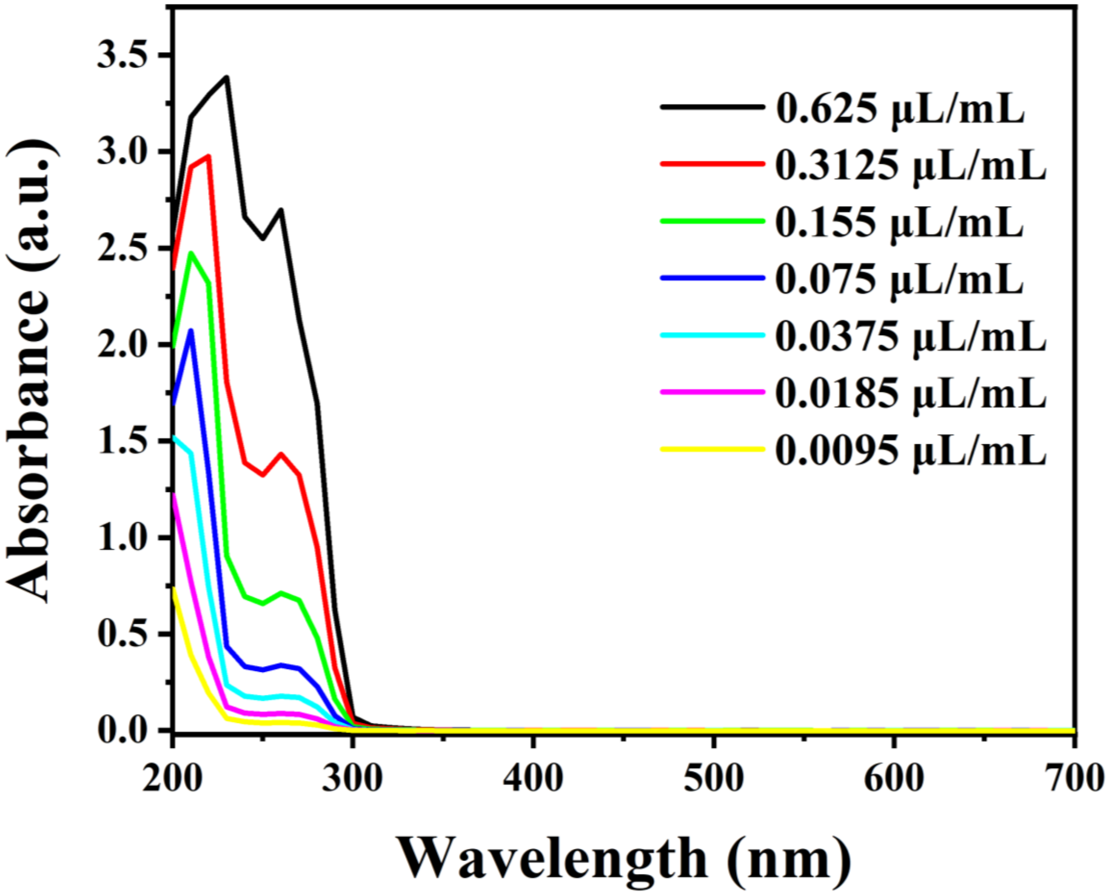

(b)

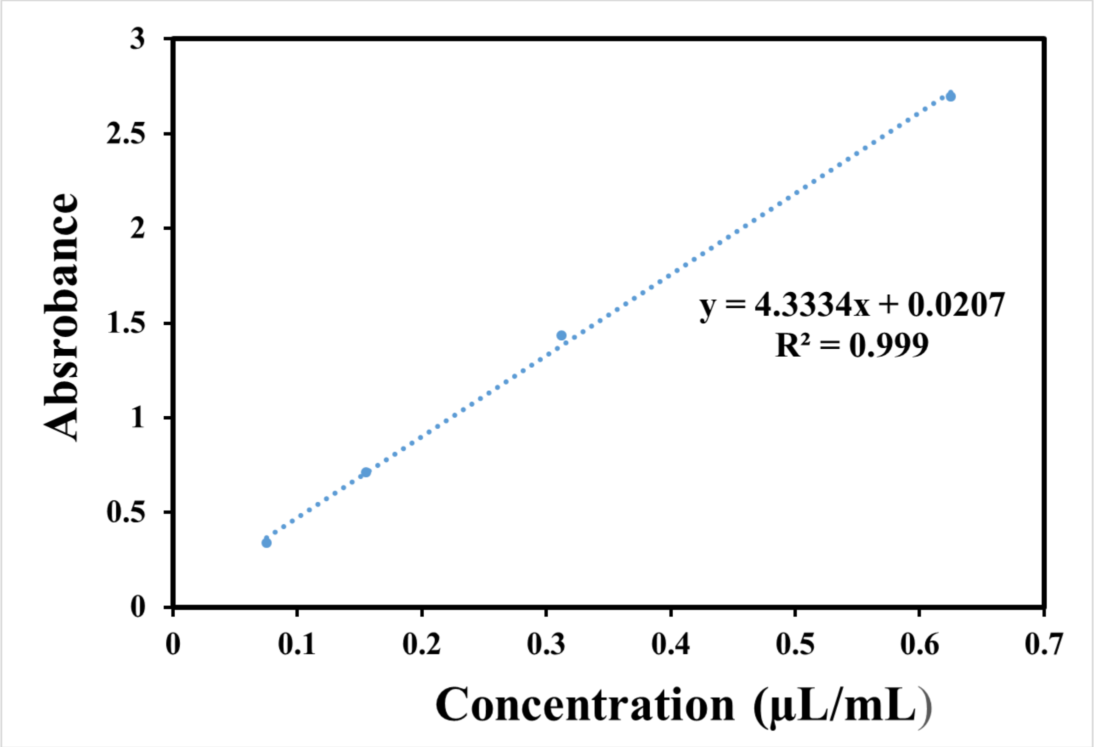

Figure S1. UV-Visible spectrum of TTO with different concentrations dissolved in hexane – a; and standard graph of TTO – b.

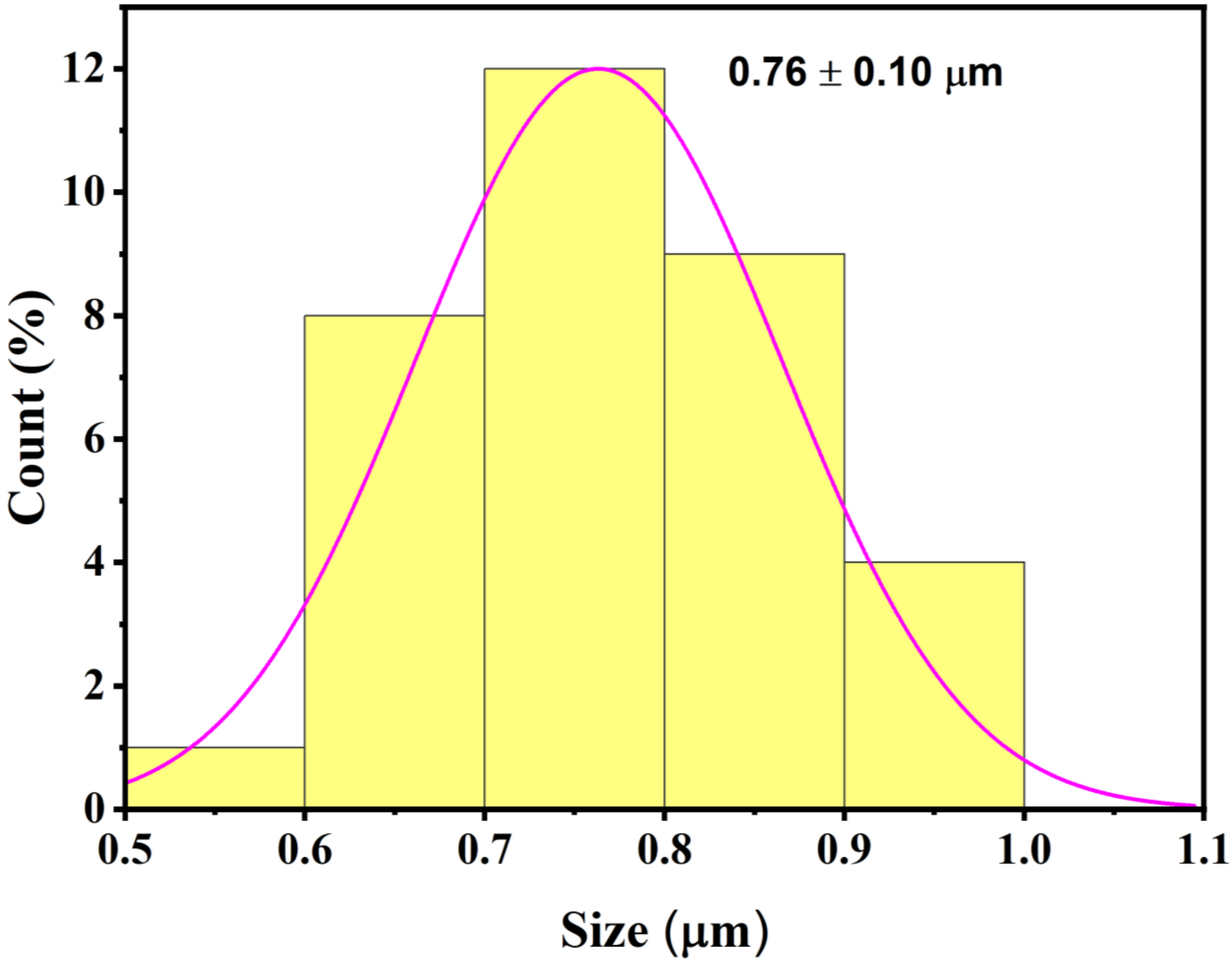

Figure S2. The particle size distribution of SA-CS-TTO microsphere determined from SEM analysis.

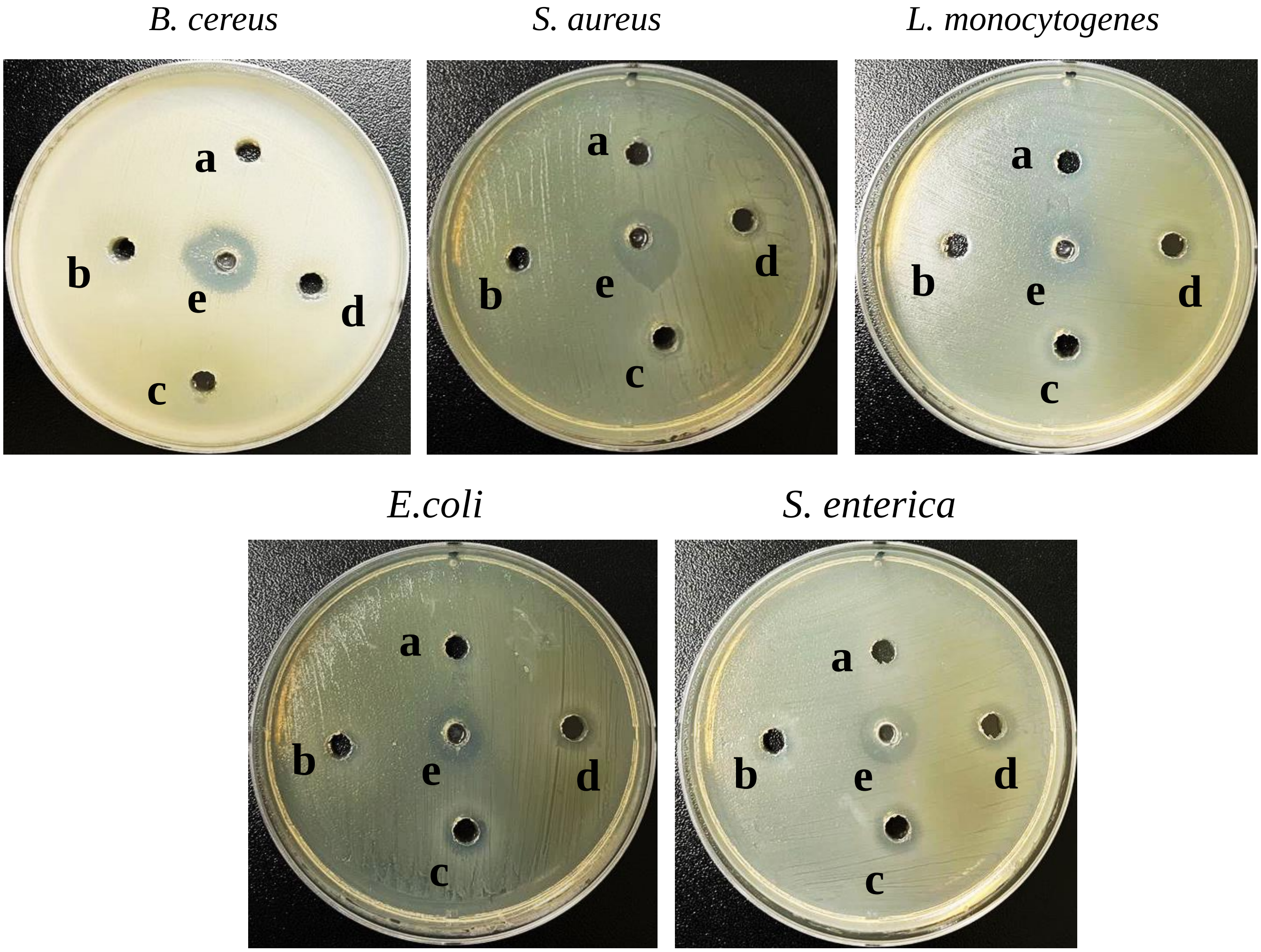

Figure S3. Antibacterial activity of CS-TTO nanoemulsion against various bacterial pathogens. a, b, c, d, and e for 5, 10, 50, 75, and 100 µg/mL.
